# Dithieno[1,4]thiazines and Bis[1]benzothieno[1,4]thiazines—Organometallic Synthesis and Functionalization of Electron Density Enriched Congeners of Phenothiazine

**DOI:** 10.3390/molecules25092180

**Published:** 2020-05-07

**Authors:** Lars May, Thomas J. J. Müller

**Affiliations:** Institut für Organische Chemie und Makromolekulare Chemie, Heinrich-Heine-Universität Düsseldorf, Universitätsstraße 1, D-40225 Düsseldorf, Germany; lars.may@hhu.de

**Keywords:** bis[1]benzothieno[1,4]thiazine, cross-coupling, dithieno[1,4]thiazine, heterocycles, Organolithium compounds, phenothiazine

## Abstract

This mini-review summarizes the syntheses and functionalizations of dithieno[1,4]thiazines and bis[1]benzothieno[1,4]thiazines, both electron density-enriched congeners of phenothiazines with remarkable electronic properties. Diversity-oriented, straightforward, and efficient syntheses, including versatile one-pot processes, have been developed for the anellated 1,4-thiazines as well as various functionalization for the expansion of the π-systems. Thereby, syntheses of different regioisomers depending on the (benzo)thieno-thiazine anellation are discussed, which exert a deep impact on the electronic properties. The tunable photophysical and electrochemical properties of dithieno[1,4]thiazines and bis[1]benzothieno[1,4]thiazines outscore phenothiazines on many points and promise an enormous potential in molecular electronics and applications beyond.

## 1. Introduction

Optimization of the properties or even the generation of new possible applications is one of the major challenges in molecular electronics [1]. Applications of electroactive materials, such as organic light-emitting diodes (OLEDs) [2], organic field-effect transistors [3,4] or photovoltaics [5,6], have continuously garnered increasing interest in the last decade. As the devices’ properties ultimately originate from the molecular properties, careful and rational design of molecular structures is crucial. Organic molecules are particularly suitable since they can be very efficiently synthesized and flexibly modified by combinatorial approaches [1,7,8]. Especially, electron-rich heterocycles adopt a pivotal role in the design concept of structures conceivable for organic electronics. Several structural motifs, including phenothiazine [9,10,11,12] and thiophene [13,14], have been extensively developed due to their favorable intrinsic electronic properties and accessibility. Phenothiazines exhibit, despite pronounced biological activity [15], fully reversible one-electron oxidations at low potentials [16], extraordinarily stable radical cations [17,18], and simultaneously luminescence, which is rather uncommon but nevertheless quite valuable for numerous applications. For instance, OLEDs [19,20,21] as well as redox switchable luminophores [10,22] have been developed based on phenothiazines. Moreover, phenothiazines are promising candidates for photovoltaics in efficient bulk-heterojunction solar cells and Grätzel-type solar cells (DSSC) [23,24,25,26,27]. Advantageously, the electronic properties can be fine-tuned by functionalization of the phenothiazine moiety, clarifying the flexible nature of organic compounds [28,29]. In addition, thiophenes, electron-rich heterocycles with tunable redox potentials, favor charge transport due to high polarizability [14]. Consequently, electron-enriched and thus highly polarizable phenothiazine analogues with promising electronic properties with regard to molecular electronics can be conceptualized upon merging phenothiazine and thiophene. The topological benzo-thieno exchange results in dithieno[1,4]thiazines **2**.

**Figure 1:**
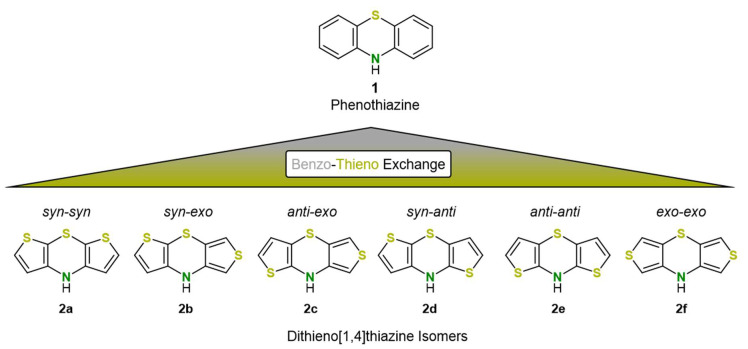
The six regioisomers **2** of the dithieno[1,4]thiazines derived from phenothiazine **1** by benzo-thieno exchange [30].

However, in contrast to benzene, thiophene can be anellated with the 1,4-thiazine in three different ways, thus leading to six dithieno[1,4]thiazine regioisomers **2** (Figure 1). For simplification, the anellation mode is abbreviated by the prefixes *syn* (2,3-*b* anellation), *anti* (3,2-*b* anellation) and *exo* (3,4-*b* anellation), depending on the orientation of the thiophene and thiazine sulfur atoms [30].

To gain access to these pronounced electron-rich congeners of phenothiazines, robust and efficient syntheses are highly desirable. This review mostly focuses on the synthesis of dithieno[1,4]thiazines and also briefly presents first insights into the electronic structure and possible applications. Furthermore, π-expanded variants of the dithieno[1,4]thiazines, bis[1]benzo-thieno[1,4]thiazines, are presented and discussed.

## 2. Early Syntheses of Dithieno[1,4]thiazines Based on Ullmann-Type Coupling

**Scheme 1:**
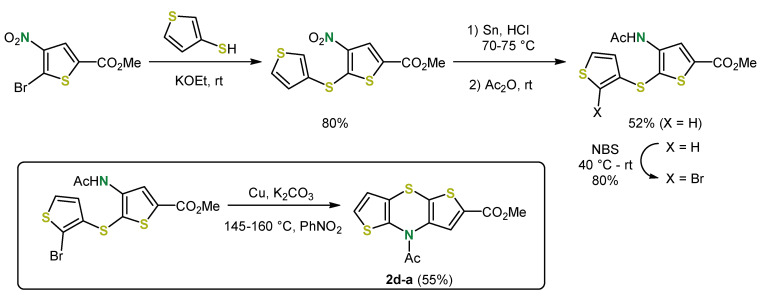
The first synthesis of a dithieno[1,4]thiazine by Grol et al.: Preparation of the *syn*-*anti* isomer **2d-a** [31].

The first synthesis of a dithieno[1,4]thiazine was reported by Grol in 1970 [31]. Since the phenothiazine moiety had been implemented in pharmaceutically active compounds, dithieno[1,4]thiazines were considered with regard to the optimization of the pharmacological properties and for expansion of the structural space. The functionalized *syn*-*anti* isomer **2d-a** was accessed in a five-step synthesis. In the first step the *syn*-*anti* orientation of the thiophene and thiazine sulfur atoms (highlighted in yellow) was introduced by nucleophilic aromatic substitution forming a 2,3′-dithienyl sulfide. Control of the regioselective nucleophilic aromatic substitution as well as introduction of the thiazine-nitrogen atom (highlighted in green) are orchestrated by a nitro group. The reduction of the latter yielded an amino dithienyl sulfide, which finally terminated in the 1,4-thiazine ring closure by an intramolecular Ullmann-type coupling after acylation and bromination (Scheme 1). Similarly, thieno[1,4]benzothiazines have been synthesized.

**Scheme 2:**
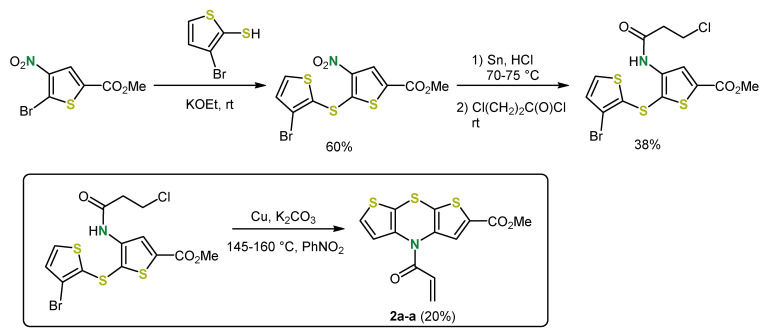
Synthesis of the *syn*-*syn* isomer **2a-a** [32].

An analogous procedure also provided the first synthesis of the functionalized *syn*-*syn* regioisomer **2a-a** [32]. As for the *syn*-*anti* isomer **2d-a**, the final anellation mode is predetermined by the position of the nitro group. With regard to further functionalization for applications as psychotropic drugs, a *β*-chloropropionic acid amide was introduced instead of the acyl group. However, due to elimination during the base-mediated Ullmann coupling, the corresponding acrylamide was formed (Scheme 2).

**Figure 2:**
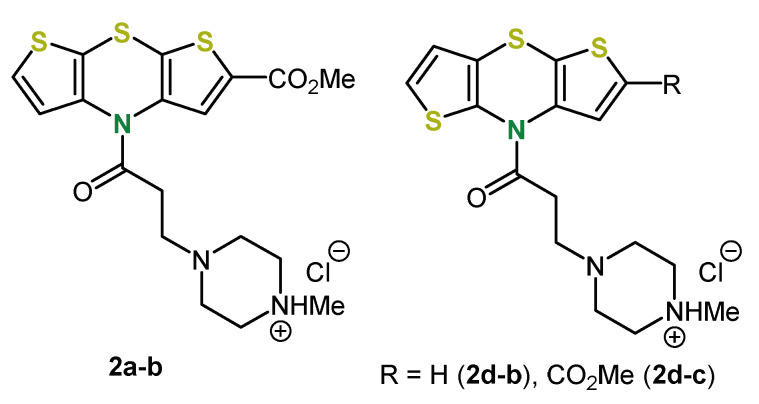
*Syn*-*syn* and *anti*-*anti* dithieno[1,4]thiazines **2a-b**, **2d-b** and **2d-c** synthesized by Michael addition [32].

In addition, by this methodology three further *N*-acryl dithieno[1,4]thiazines **2a-b**, **2d-b** and **2d-c** were synthesized, which were further functionalized by a Michael addition of *N*-methylpiperazine (Figure 2). However, reducing the carbonyl group was not successful, so that according to Grol the intended pharmacological activity could not be achieved [32].

**Scheme 3:**
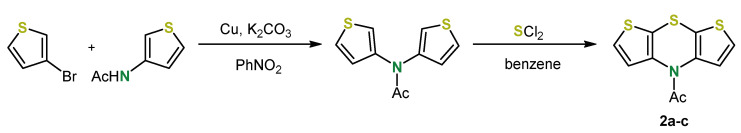
Concise two-step synthesis of the *N*-acyl *syn*-*syn* dithieno[1,4]thiazine **2a-c** [33].

In the case of *syn*-*syn* isomers **2a** the rather time-consuming synthesis according to Grol (Scheme 2) was cut short by an alternative strategy for generating the 1,4-thiazine ring. At first, introducing the thiazine nitrogen bridge by an Ullmann-type coupling and then closing the thiazine ring by cyclizing electrophilic aromatic substitution with sulfur dichloride gave dithieno[1,4]thiazine **2a-c** (Scheme 3) [33]. Cleavage of the acyl-group, setting the stage for further *N*-functionalization by both acidic and basic hydrolysis, was not successful.

## 3. Syntheses of Dithieno[1,4]thiazines by Cyclizing Buchwald–Hartwig Coupling

**Scheme 4:**
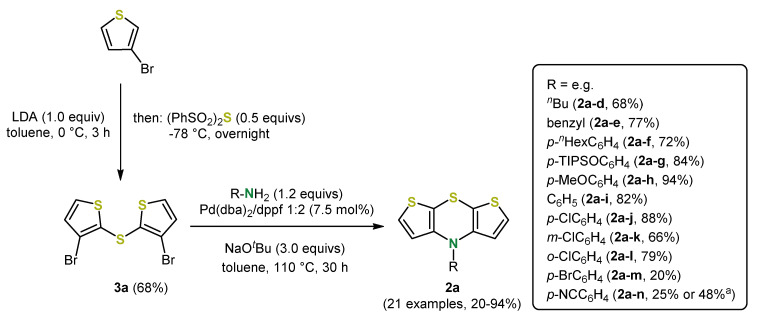
Diversity-oriented synthesis of *syn*-*syn* dithieno[1,4]thiazines 2a via inter-intra-molecular Buchwald-Hartwig coupling; a: Dielectric heating at 160 °C [35,36,37].

Inspired by Jørgensen’s phenothiazine synthesis (palladium-catalyzed formation of the sulfur and nitrogen bridge) [34], a more diversity-oriented and straightforward synthesis of *N*-functionalized *syn*-*syn* dithieno[1,4]thiazines **2a** via cyclizing Buchwald–Hartwig coupling was conceived and developed by Dostert (Scheme 4) [35]. For the synthesis of the *syn*-*syn* dithieno[1,4]thiazines, sodium *tert*-butoxide as a base and dibenzylidenepalladium(0) and 1,1′-bis(diphenylphosphano)ferrocene (dppf) as a catalyst in toluene were used by default.

**Scheme 5:**
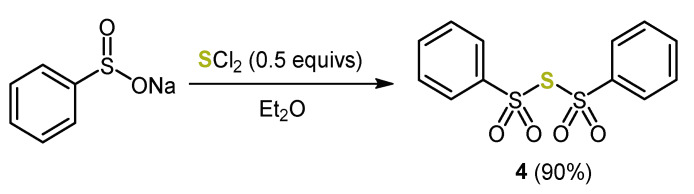
Synthesis of bis(phenylsulfonyl)sulfide **4** [39].

In contrast to earlier syntheses, the 1,4-thiazine-precursor, the dithienyl sulfide **3a**, is conveniently generated starting from commercially available 3-bromo thiophene [38]. The efficient introduction of the sulfur bridge was achieved by double nucleophilic substitution at bis(phenylsulfonyl)sulfide **4**, which was synthesized from sulfur dichloride (Scheme 5) [39].

By palladium-catalyzed inter- and intramolecular Buchwald–Hartwig coupling a broad spectrum of *N*-functionalized *syn*-*syn* dithieno[1,4]thiazines **2a** is accessible, which was illustrated by 18 examples. Electron-withdrawing (e.g., **2a-n**) or electron-donating (e.g., **2a-h**) *N*-aryl substituents and also *N*-benzyl (**2a-e**) and *N*-alkyl substituents (e.g., **2a-d**) were implemented with mostly good to very good yields (20%–94%). The yield of dithieno[1,4]thiazines bearing strong acceptor substituents like cyano groups can be raised by dielectric heating at 160 °C. *Ortho*- and *meta*-chloro substituents can be present as well. It should be particularly emphasized that *p*-bromophenyl was introduced with a moderate yield of 20%, which could, for example, serve as a starting point for further cross-coupling [35].

**Scheme 6:**
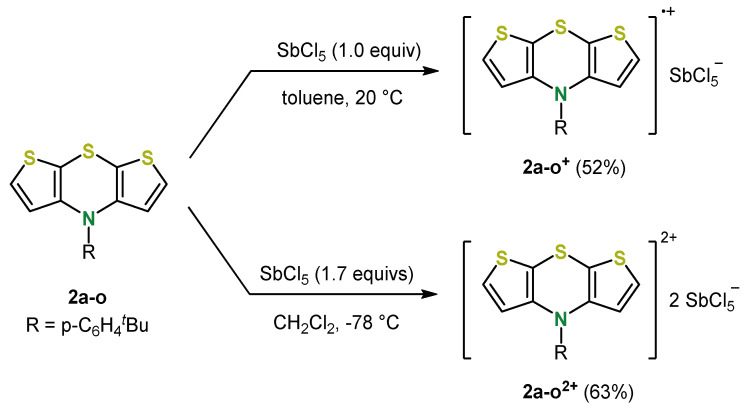
Synthesis of stable the radical cations **2a-o^+^** and dication **2a-o^2+^** [40].

This diversity-oriented, robust and therefore efficient synthesis allowed a detailed characterization of the electronic properties and structure of the *N*-functionalized *syn*-*syn* dithieno[1,4]thiazines in comparison to phenothiazines. While the structure of dithieno[1,4]thiazines expectedly corresponds to that of phenothiazines, the electronic properties differ quite remarkably in some points. For example, the oxidation potential of *N*-phenyl *syn*-*syn* dithieno[1,4]thiazine (**2a-i**) is strongly shifted cathodically by about 320 mV compared to *N*-phenyl phenothiazine, so that *syn*-*syn* dithieno[1,4]thiazines **2a** could actually be verified as electron-rich phenothiazine analogues with respect to the electronic properties. In addition, the oxidation potentials of *syn*-*syn* isomers **2a** can be widely tuned by the electron-withdrawing or electron-donating character of the *N*-substituents [35]. Moreover, the outstanding stabilization of the radical cations formed by oxidation, typical for phenothiazines [17] but even more pronounced for *syn*-*syn* dithieno[1,4]thiazines **2a**, allows the isolation and characterization of mono- and dioxidized *syn*-*syn* dithieno[1,4]thiazines. The radical cation **2a-o^+^** and the dication **2a-o^2+^** were prepared by oxidation with antimony(V)chloride (Scheme 6) [40].

EPR (electron paramagnetic resonance) measurements of **2a-o^+^** suggest a high spin density on the thiazine nitrogen atom but also a noticeable delocalization on the dithieno[1,4]thiazine system, whereas the dication **2a-o^2+^** is EPR silent but NMR active suggesting an Hückel-aromatic singlet state [40].

**Scheme 7:**
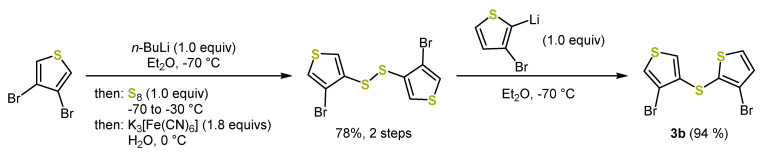
Multi-step synthesis of the unsymmetrical 2,3-dithienyl sulfide **3b**. The key step is the use of a symmetrically substituted dithienyl disulfide, which reacts as a sulfur monoelectrophile with thienyl lithium and, thus, desymmetrizes [41].

To access further dithieno[1,4]thiazine isomers **2**, the synthesis of appropriate dithienyl sulfides **3** was a prerequisite. Symmetric 2,2′- and 3,3′-dithienyl sulfides like **3a**, **3e**, or **3f** have been synthesized using bis(phenylsulfonyl)sulfide **4** [38,41]. However, more complex multistep syntheses (Scheme 7) were required for unsymmetrically substituted dithienyl sulfides, such as **3b** [41]. This was a major disadvantage, in particular with regard to the preparation time and atom economy.

**Scheme 8:**
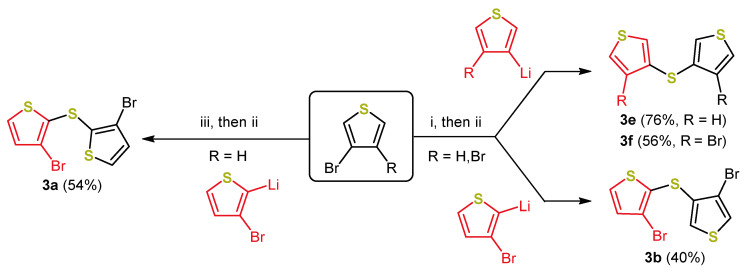
One-pot synthesis of **3a**, **3b**, **3e**, and **3f**: (**i**) 1.0 equiv *n*-BuLi, Et_2_O, −78 °C; then: 1.0 equiv S_8_, −78 to 0 °C; then: 1.0 equiv *p*-TsCl, 0 to 40 °C; (**ii**) 1.2 equivs thienyllithium derivative, −78 °C; (**iii**) 1.0 equiv LDA, Et_2_O, −78 °C; then: 1.0 equiv S_8_, −78 to 0 °C; then: 1.0 equiv *p*-TsCl, 0 to 40 °C [30].

A synthetic approach towards dithienyl sulfides **3** based on a multicomponent reaction [7] tackled this issue. Chen et al. presented a consecutive three-component reaction for the synthesis of dithieno[2,3-*b*:3′,2′-*d*]thiophenes with good yields in a one-pot fashion [42]. In this process, elemental sulfur itself acts as an electrophile, which renders the use of **4** as unnecessary, and thus raised the atom economy and reduced the preparation time drastically. Moreover, in contrast to earlier syntheses, the use of elemental sulfur as well (Scheme 7), further oxidations and isolation steps are circumvented. Modifying this one-pot sequence opened new avenues for the synthesis of both symmetrically and unsymmetrically substituted dithienyl sulfides in a divergent one-pot fashion. Starting from commercially available bromo thiophenes, three isomeric dithienyl sulfides (**3b**, **3e**, and **3f**) were synthesized in addition to the *syn*,*syn*-dithienyl sulfide **3a** with good to very good yields (Scheme 8) [30].

**Figure 3:**
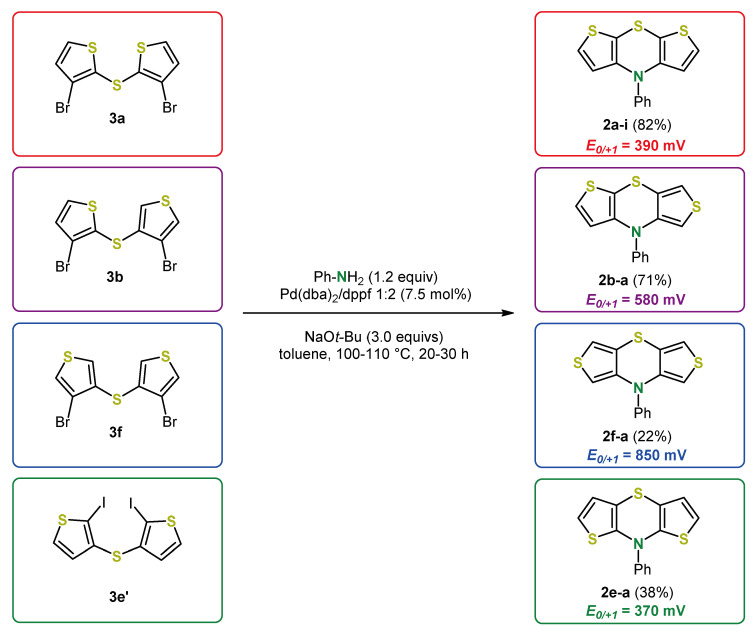
Synthesized dithieno[1,4]thiazine regioisomers **2** and corresponding redox potentials *E_0/+1_* (in CH_2_Cl_2_, *T* = 298 K, vs. decamethyl ferrocene) [30,35].

The yields of this one-pot sequence were on the same scale as the overall yields of the stepwise processes (Scheme 7) and the desired dithienyl sulfides were provided in just one step [30,35,41]. Only in the case of 3,3′-dithienyl sulfide **3e** an additional iodination (86% yield of **3e′**; for the structure, see Figure 3) has to be performed. The *syn*-*exo*, *exo*-*exo* and *anti*-*anti* dithieno[1,4]thiazine isomers **2b-a**, **2f-a**, and **2e-a** bearing a *N*-phenyl substituent were analogously synthesized to the *syn*-*syn* isomers **2a** (Scheme 4) by Buchwald–Hartwig coupling. However, the yields dropped compared to the *syn*-*syn* isomers (Figure 3) [30,35].

According to the DFT (density functional theory) calculations and X-ray analysis (X-ray only for **2-i**) the dithieno[1,4]thiazines 2 are folded along the *S,N*-axis resulting in an phenothiazine-like butterfly structure [30,35]. The electronic properties are highly dependent on the anellation mode. For example, the oxidation potential of the *anti*-*anti* isomer **2e-a** is even more cathodically shifted than for the *syn*-*syn* isomer **2a-i**. In contrast, the oxidation potential of the *exo*-*exo* isomer **2f-a** is more anodically shifted than for **2a-i** because of a less efficient delocalization of the π-electron density. Whereas the *syn*-*syn* isomers **2a** fluoresce very weakly [35], which is atypical for comparable phenothiazines [43], the *anti*-*anti* isomer **2e-a** fluoresces rather phenothiazine-like [30].

## 4. 2,6-Functionalization of Dithieno[1,4]thiazines: Expanding the π-Systems

**Scheme 9:**
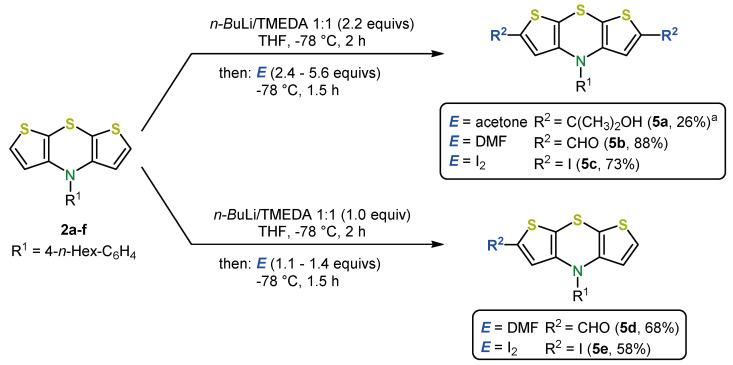
Synthesis of 2,6-di- and 2-monofunctionalized *syn*-*syn* dithieno[1,4]thiazines **5** via dilithiation and electrophile trapping (*E* = electrophile); a: R^1^ = Ph [36,37].

Functionalization of the dithieno[1,4]thiazine moiety can be directly achieved by straightforward lithiation-electrophile trapping sequences and does not require a preceding halogenation in contrast to phenothiazines (Scheme 9). *α*-Lithiation of thiophenes using *n*-butyllithium sets the stage for various 2,6-functionalization. Thereby, di- as well as monofunctionalizations are readily realized [36,44,45].

Important dithieno[1,4]thiazine building blocks **5**, for example, aldehydes, iodides, or alcohols, are accessible with mostly good yields using dimethylformamide (DMF), iodine, or acetone as electrophiles, respectively [36,37].

**Scheme 10:**
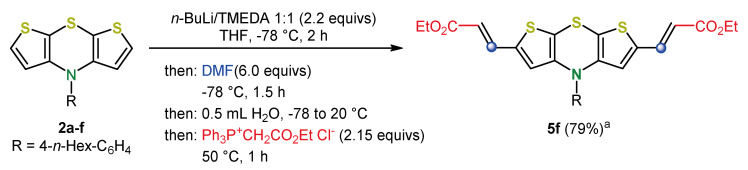
Synthesis of the π-expanded dithieno[1,4]thiazine **5f** in the sense of a pseudo five-component formylation-Wittig sequence via post-functionalization of *syn*-*syn* **2a-f**; a: 58% overall-yield in a two-step synthesis isolating the aldehyde intermediate [36].

These building blocks set the stage for a diverse range of follow-up chemistry. For example, starting from aldehyde **5b**, expanded π-conjugated systems are conceivable via Wittig reactions in good yields. Moreover, the Wittig reaction could be implemented in a one-pot sequence starting from 2,6-unsubstituted dithieno[1,4]thiazine **2a-f**, circumventing the isolation of the intermediary aldehyde. This pseudo-five-component reaction is clearly superior to the stepwise synthesis due to the shortened preparation time and increased yield (Scheme 10) [36].

**Scheme 11:**
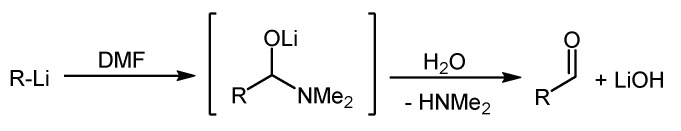
Concatenation of formylation and Wittig reaction by exploiting the basicity generated during the preceding formylation [46].

Concatenating formylation with the Wittig reaction was inspired by a method reported by Schlosser [46]. The basic milieu initially originating from the organo lithium is transferred across the formylation and is finally further exploited in the Wittig reaction, so that no additional base has to be added for the generation of the ylide (Scheme 11).

**Scheme 12:**
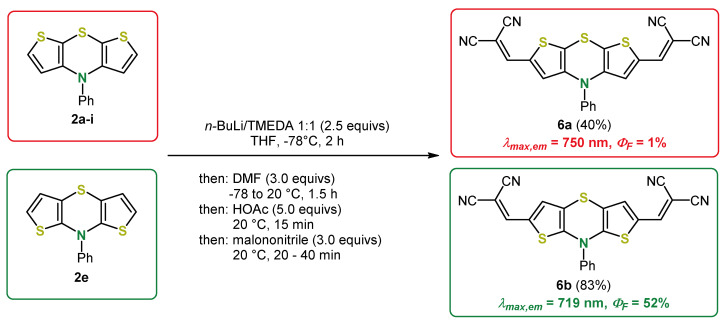
Pseudo five-component one-pot synthesis of 2,6-diacceptor substituted *syn*-*syn* and *anti*-*anti* dithieno[1,4]thiazines **6a** and **6b** via lithiation-formylation-Knoevenagel sequence [45].

Other one-pot sequences, similarly based on a lithiation-formylation sequence, focus on the use of dimethyl amine, which is present in equilibrium after the formylation, as an organocatalyst for subsequent Knoevenagel condensations [47]. Hence, dithieno[1,4]thiazines were also efficiently functionalized via a lithiation-formylation-Knoevenagel sequence in a one-pot fashion (Scheme 12) [45].

The dithieno[1,4]thiazine-acceptor conjugates **6** exhibit anodically shifted redox potentials and strongly bathochromically shifted UV/vis absorption bands with a charge transfer character, which are accompanied by intense colors. By eyesight, the aldehydes already appear deep red, and with the stronger acceptors as implemented via Knoevenagel condensation, deep blue chromophores are formed. In contrast to *syn*-*syn* isomers, the fluorescence quantum yields *Φ_F_* of *anti*-*anti* isomers are remarkably high and in the order of the corresponding phenothiazines (*λ_max,em_* = 639 nm, *Φ_F_* = 57%). Besides even more bathochromically and furthermore hyperchromically shifted absorption bands, the emission of the acceptor-substituted *anti*-*anti* isomers is intensified compared to the *syn*-*syn* isomers. Ultimately, dithieno[1,4]thiazine **6b** exhibited an outstanding intense NIR fluorescence at *λ_max,em_* = 719 nm (*Φ_F_* = 52%), whereas **6a** fluoresces significantly less intensively (*λ_max,em_* = 750 nm, *Φ_F_* = 1%). [45].

**Scheme 13:**
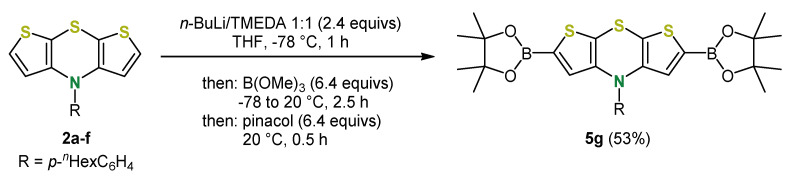
Synthesis of dithieno[1,4]thiazine-based boronic acid esters [37].

In addition, borylation of lithiated dithieno[1,4]thiazines can be carried out with trimethylborate as an electrophile, whereby pinacol-boronic acid esters, versatile substrates for Suzuki couplings, were obtained after subsequent esterification (Scheme 13) [37].

**Scheme 14:**
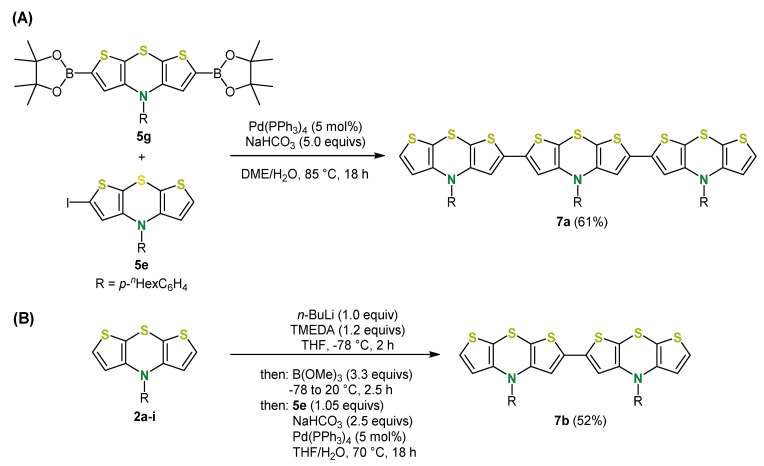
(**A**) Synthesis of dithieno[1,4]thiazine-trimer **7a**; (**B**) synthesis of dithieno[1,4]thia-zine-dimer **7b** [37].

Suzuki coupling of the borylated and halogenated dithieno[1,4]thiazine building-blocks **5g** and **5e** furnish dithieno[1,4]thiazine trimers **7a** (Scheme 14A). For the synthesis of dimers **7b**, another one-pot sequence, namely a borylation-Suzuki coupling sequence, was developed circumventing the isolation of borylated intermediates (Scheme 14B). In contrast to the monomers **2a-f**, these oligomers fluoresce redshifted and more intensively [37].

**Scheme 15:**
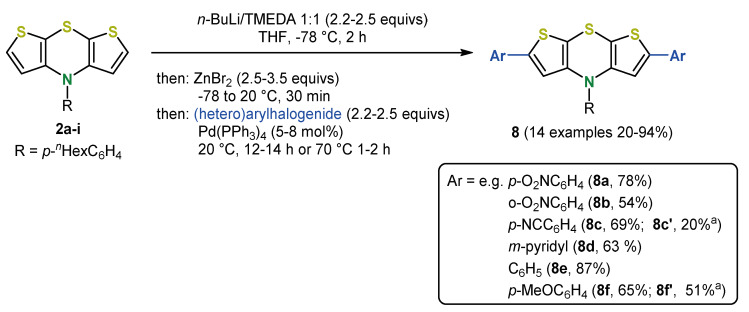
Selected direct arylations of dithieno[1,4]thiazines via one-pot lithium-zinc exchange-Negishi coupling; a: R = Ph [44,45].

Moreover, Negishi coupling is also perfectly suited for direct arylations of *syn*-*syn* dithieno[1,4]thiazines **2a** [44] and can be alternatively employed for the synthesis of dimer **7b** [37]. Using zinc dibromide as a trapping electrophile for the lithiated dithieno[1,4]thiazine species, the resulting organozinc intermediate can be reacted with various arylhalides in the sense of a Negishi coupling. With aryliodides, the reaction already proceeds smoothly at room temperature with good to very good yields. Besides electron-donating (hetero)aryl substituents, electron-withdrawing substituents can be introduced and *ortho*-substituents are also tolerated (Scheme 15) [37]. In addition, monoarylations and the diarylation of the *anti*-*anti* dithieno[1,4]thiazines **2e** proceed similarly [44,45]. Polymerization of *syn*-*syn* dithieno[1,4]thiazines was accomplished by polymer analogous Negishi couplings using a diiodide as the dihalogen substrate. The polymer reaches a degree of polymerization of 37 (*M_w_*/*M_n_* = 1.32) and a significantly lower band gap than oligophenothiazines can be determined by spectroscopy and computations. Therefore, dithieno[1,4]thiazine oligomers and polymers are potentially interesting as *p*-type semiconductors in organic electronics, similar to phenothiazines [48].

Similar to substituents in the *N*-position, the substituents in 2,6-positions can exert a great influence on the photophysical and electrochemical properties. However, 2,6-substituted *syn*-*syn* dithieno[1,4]thiazines mostly do not fluorescence intensely, whereas the *anti*-*anti* isomers do indeed. Furthermore, the π-system of the *anti*-*anti* isomers interacts with the π-system of the substituents by full conjugation more efficiently than in *syn*-*syn* isomers triggering the different electronic properties of the isomers [45]. Substituents in the 2,6-positions have a stronger influence on the redox potentials than the substituents in the *N*-position, which was demonstrated for *syn*-*syn* isomers by a different response to the substituent effects (correlation with Hammett parameters) [35,36]. With donor substituents, the oxidation potential of the 2,6-diarylated dithieno[1,4]thiazines **8** decreases, whereas acceptor substituents cause an increase. However, diarylation seems to reduce the stabilization of the radical cations formed by oxidation [44,45], but they still remain at a relatively high level compared to other redox active π-systems [17]. In addition, the lowest energy absorption bands strongly shift bathochromically with an increasing acceptor strength [44,45].

## 5. Expansion of the π-System by Anellation: Bis[1]benzothieno[1,4]thiazines

**Scheme 16:**
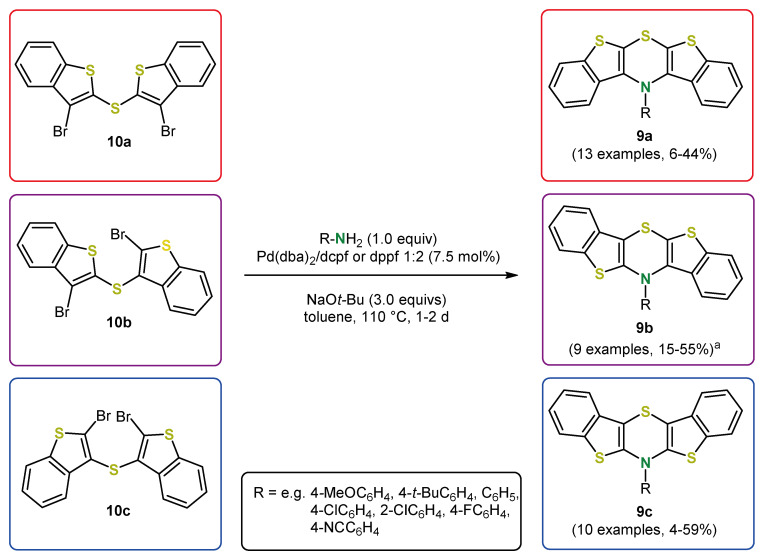
Synthesized bis[1]benzothieno[1,4]thiazine regioisomers **9** (^a^ Additional examples of **9b** were obtained as side products in the synthesis of *syn*-*syn* isomers **9a**) [49,50].

Thieno expansion rather than benzo-thieno replacement in phenothiazines formally lead to bis[1]benzothieno[1,4]thiazines **9**, which can also be considered as benzo anellated dithieno[1,4]thiazines. There are three regioisomers, the *syn*-*syn*, the *syn*-*anti* and the *anti*-*anti* isomer (Scheme 16). Their synthesis is generally in accordance with the synthesis of the dithieno[1,4]thiazines **2** (Scheme 4) [49,50]. A broad spectrum of *N*-functionalization, including electron-withdrawing and electron-donating *N*-aryl substituents, is also accessible. *Ortho* substituents on the *N*-aryl components are generally introduced with a slight drop in yield. The yields are mostly moderate but are lower for *syn*-*syn* isomers, presumably due to steric hindrance.

**Scheme 17:**
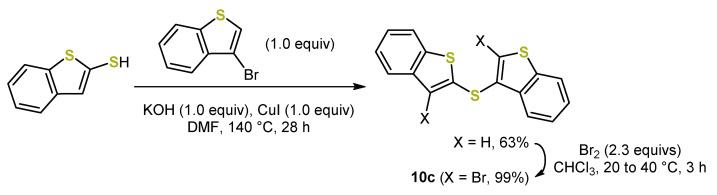
Synthesis of the benzo-anellated 2,3-dithienyl sulfide **10c**. In preparation of the Buchwald–Hartwig coupling towards the *syn*-*anti* bis[1]benzothieno[1,4]thiazines **9c**, a bromination had to be included [50].

The benzo-anellated 2,2′- and 3,3′-dithienyl sulfides **10** have been similarly synthesized to the dithienyl sulfides by introducing the sulfide bridge using bis(phenylsulfonyl)sulfide **4** (Scheme 5). However, the 2,3-dithienyl sulfide **10c** has been prepared via an Ullmann-type *CS*-coupling (Scheme 17) [50].

**Scheme 18:**
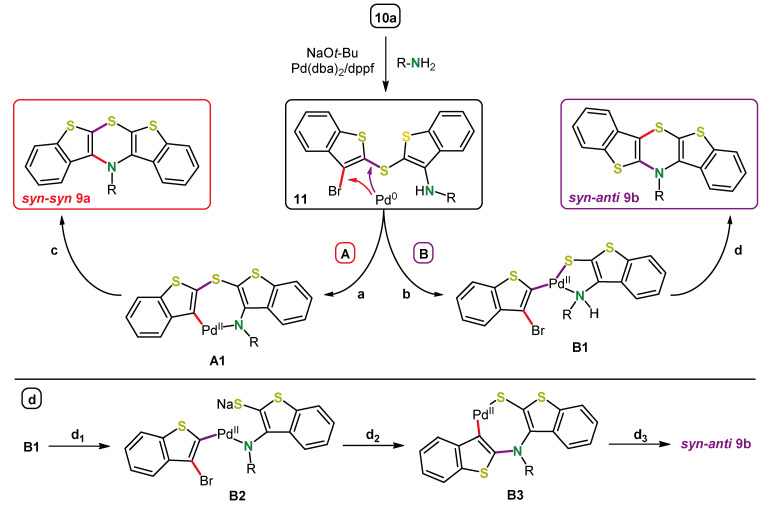
Condensed plausible mechanistic rationale of the *syn*-*anti* isomerization. Pathways A and B generate the *syn*-*syn* or the *syn*-*anti* isomer respectively. a) Oxidative addition of Pd into the *CBr*-bond, coordination and deprotonation of the amine; b) Oxidative addition of Pd into the *CS*-bond and coordination of the amine; c) Reductive elimination of Pd forming *CN*-bond; d_1_) Deprotonation of the amine and sulfide dissociation from Pd; d_2_) Reductive elimination of Pd forming *CN*-bond, then oxidative addition of Pd into the *CBr*-bond, coordination of the sulfide; d_3_) Reductive elimination of Pd forming *CS*-bond [49].

The choice of ligand was crucial for obtaining selectivity. In some cases, an isomerization occurred during Buchwald–Hartwig coupling using dppf as a ligand. For example, starting from *syn*-*syn* bis(3-bromobenzo[b]thiophen-2-yl)sulfane (**10a**), a 3:1 mixture of the *syn*-*syn* and the *syn*-*anti* bis[1]benzothieno[1,4]thiazines **9a** and **9b** was obtained using dppf [49]. Bulkier ferrocene ligands like 1,1′-bis-(dicyclohexylphosphano)ferrocene (dcpf) or 1,1′-bis(di-*tert*-butylphosphano)ferrocene (dtbpf) decreased the isomerization significantly. In addition, the increased donor strength of dcpf and dtbpf compared to dppf seemed to be beneficial in terms of selectivity. For the *syn*-*syn* and the *syn*-*anti* isomers, dcpf gave the highest yields and a selective coupling without isomerization, while for the *anti*-*anti* isomers, dppf already gave reasonable results. Based on the evidence of an intermediate, namely the product of the intermolecular Buchwald–Hartwig coupling **11**, and a kinetic study on the formation of intermediates a mechanistic rationale for the *syn*-*anti* isomerization was derived (Scheme 18). The keystep is the site-selective oxidative addition into either the *CBr*- or the *CS*-bond of intermediate **11**, which then follows a usual Buchwald–Hartwig-type mechanism (path A) or an alternate isomerizing reaction cascade (path B), respectively. The influence of the bulkiness of the ligand on the product distribution suggests a kinetically favored generation of *syn*-*syn* isomers, whereas *syn*-*anti* isomers seem to be thermodynamically favored according to the influence of the reaction temperature. Similarly to dithieno[1,4]thiazines **2**, the photophysical and electrochemical properties of bis[1]benzothieno[1,4]thiazines **9** strongly depend on the anellation mode and can thereby be tuned and in addition by *N*-substitution. On the one hand, the oxidation potentials are slightly increased compared to the dithieno[1,4]thiazines; nevertheless they are still below phenothiazines. However, on the other hand, the fluorescence quantum yields are increased. Bis[1]benzothieno[1,4]thiazines **9** not only fluoresce in solution but also in the solid state, depending on their conformation, which, in turn, can be controlled by the anellation mode and the substitution. Ultimately, a rather unique flattened conformation of the 1,4-thiazine ring is adopted in the solid state, for example, for bis[1]benzothieno[1,4]thiazine **9c** (R = *t*-Bu) [49,50]. In addition, the electronic properties in the solid state (redshifted fluorescence) and NICS (nucleus-independent chemical shift) calculation strongly support the antiaromaticity of the fully planarized conformation of *anti*-*anti* bis[1]benzo-thieno[1,4]thiazines **9c** prevalent in the solid state.

## 6. Conclusions

Dithieno[1,4]thiazines and their benzo anellated congeners, bis[1]benzothieno[1,4]thiazines, can be synthesized by straightforward and efficient Buchwald–Hartwig coupling starting from primary amines and dithienyl sulfides or the corresponding benzo-anellated dithienyl sulfides. In addition, these sulfide precursors are efficiently accessible from commercially available bromo thiophenes, eventually in a one-pot fashion, also furnishing unsymmetrically substituted dithienyl sulfides in just a single operation. Unlike phenothiazines, there are several regioisomers depending on the (benzo)thieno anellation. All these isomers have been synthesized for bis[1]benzothieno[1,4]thiazines and most of them for dithieno[1,4]thiazines by applying cyclizing Buchwald–Hartwig coupling. Only the *syn*-*anti* isomer has been exclusively synthesized via a multistep route completed by an Ullmann-type coupling. The electronic properties decisively depend on the anellation mode and oxidation potentials are varied in a broad range by just altering the (benzo)thieno anellation. Hence, dithieno[1,4]thiazines and bis[1]benzothieno[1,4]thiazines exhibit broad intrinsic electronic diversity, which is almost completely addressable by the preparation. Moreover, dithieno[1,4]thiazines and bis[1]benzothieno[1,4]thiazines generally outscore by electron enrichment the oxidation potentials of phenothiazines.

Besides the choice of isomer itself, functionalization significantly controls the electronic properties. Various implemented *N*-functionalizations not only tune the oxidation potentials or the molecular geometry but also the photophysical properties. For instance, planarized, stable antiaromatic bis[1]benzothieno[1,4]thiazines become accessible by variation of *N*-substituents. Especially, functionalization of the *α*-positions of dithieno[1,4thiazines exerts an enormous impact on the photophysical properties, as indicated by the remarkably intense NIR fluorescence of a 2,6-acceptor-substituted *anti*-*anti* dithieno[1,4]thiazine. Manifold functionalization of the *α*-positions relies on lithiation-electrophile-trapping one-pot methodology providing useful building blocks, eventually also opening by transmetalation access to novel consecutive multicomponent reactions. Most interestingly, dithieno[1,4]thiazines and bis[1]benzothieno[1,4]thiazines solicit themselves as phenothiazine substitutes. The tunable intense absorption realized on a relatively small molecular dimension by the enhanced strong donor properties of dithieno[1,4]thiazines suggest an application as absorbers in the active layer of organic solar cells, where phenothiazines have already been applied [24]. Furthermore, electroactive emitters like the presented dithieno[1,4]thiazines appear as potential candidates for efficient OLEDs [45], as already shown for phenothiazines.[21] In particular, intense NIR emission is increasingly interesting for biomedical imaging [51]. Oligomers and polymers of the dithieno[1,4]thiazines fulfill critical requirements as *p*-type semiconductors [37]. In conclusion, (benzo)thieno-anellated 1,4-thiazines as electron-richer congeners of phenothiazines have opened new avenues for novel functional π-electron systems with exciting properties both for fundamental and applied research.
